# Exploring the determinants of midwives’ perinatal bereavement support behaviour to mothers using the theory of planned behaviour in the Upper West Region of Ghana

**DOI:** 10.1371/journal.pone.0344531

**Published:** 2026-03-18

**Authors:** Veronica Sonasal Dooh, Evelyn Asamoah Ampofo, Adiza Atoko Mumuni, Michael Darko Ashaley, Mary Ani-Amponsah, Louis Nebayeng Mornah

**Affiliations:** 1 School of Nursing and Midwifery, University of Ghana, Accra, Ghana; 2 Biostatistics unit, Department of Surgery, Korle-Bu Teaching Hospital, Accra, Ghana; 3 Department of Nursing and Midwifery Garden city, University Kumasi, Ghana; Chinese University of Hong Kong, HONG KONG

## Abstract

**Background:**

Perinatal loss presents profound emotional challenges requiring skilled bereavement support from midwives. Despite its importance, determinants of midwives’ bereavement support behaviour remain underexplored in low-resource settings. Using the constructs in the Theory of Planned Behaviour, this study examined factors influencing midwives’ bereavement support practices in Ghana’s Upper West Region. This study examined determinants of midwives’ behaviour in providing bereavement support to mothers experiencing perinatal loss in the Upper West Region.

**Methods:**

A cross-sectional study was conducted among 269 registered midwives (response rate = 98%) across selected health facilities. Data were collected using structured questionnaires based on the Theory of Planned Behaviour (TPB). Analyses were performed in SPSS version 27.0 and AMOS, employing descriptive statistics, correlation analysis, and path analysis with bootstrapping to examine predictors of bereavement support behaviour.

**Results:**

Among 269 midwives (response rate = 98%; mean age = 34 years), attitudes (M = 3.26, SD = 0.41) and perceived behavioural control (M = 3.02, SD = 0.57) were generally favourable. Path analysis revealed that attitude (β = 1.00, p < .001) and perceived behavioural control (β = .32, p < .001) significantly predicted behavioural intention, which strongly predicted actual behaviour (β = .65, p < .001). Notably, subjective norms had a negative effect (β = −.21, p < .001), and bereavement training were not statistically significant predictors of midwives’ bereavement support behaviour.

**Conclusion:**

Midwives demonstrated favourable attitudes toward bereavement support, yet actual practice was primarily driven by personal attitudes and perceived control rather than training or social norms. To improve bereavement care, health systems should build midwives’ confidence through experiential training and mentorship, establish institutional cultures that normalize bereavement support, and integrate bereavement care protocols into routine maternal health services. These evidence-based strategies can enhance compassionate support for mothers experiencing perinatal loss in resource-limited settings.

## Introduction

Perinatal loss represents one of the most distressing and emotionally devastating experiences for mothers and families worldwide [1–4]. Defined as the loss of a foetus or newborn from the period of viability (around 22 weeks of gestation) through the first week of life [5,6], perinatal loss remains a significant contributor to global child mortality [7]. It may occur due to wilful termination of pregnancy or intrapartum death, miscarriage, late miscarriage, and ectopic pregnancy [8]. In 2021 alone, about 2.3 million babies died within their first month of life, accounting for nearly 47% of all under-five deaths globally [9]. In Sub-Saharan Africa, where healthcare systems often face resource and infrastructural challenges, the burden is even more pronounced, with one in every thirty-six babies dying within the neonatal period compared to one in every three hundred and thirty-three in developed regions [10,11]

In Ghana, perinatal loss continues to be a major public health concern. As of 2019, the neonatal mortality rate stood at 23.1 deaths per 1,000 live births, while the stillbirth rate was 23 per 1,000 total births in 2017 [12]. These losses are not only medical or statistical events but deeply personal tragedies that often leave profound psychological, emotional, and social scars on affected mothers and families. Studies have linked perinatal loss with increased risk of depression, anxiety, post-traumatic stress disorder, and even suicide [2,13,14]. The grieving process is further complicated by cultural beliefs, inadequate emotional support systems, and poor communication with healthcare providers, especially in many low- and middle-income countries, including Ghana [12,15].

Midwives, as frontline maternal health providers, are uniquely positioned to offer bereavement support to mothers following perinatal loss. Their behaviour and attitudes during such critical moments can profoundly influence the emotional recovery and long-term wellbeing of bereaved women. Compassionate communication, empathetic care, and appropriate counselling can alleviate the psychological burden, whereas negative interactions may intensify feelings of grief and isolation. However, research shows that midwives in low-resource settings often feel ill-equipped to provide bereavement care due to lack of training, limited institutional support, and the absence of clear guidelines [16,17].

Despite the critical role midwives play, there is limited evidence on what determines their behaviour in providing bereavement support, particularly in the Upper West Region which faces significant socio-cultural complexity and health system constraints. Midwives may face various personal, organizational, and societal barriers that influence their willingness and ability to support grieving mothers. Understanding these determinants is essential to strengthening maternal health care and ensuring respectful, compassionate responses to perinatal loss. [12,16]. There is currently limited evidence on the skills and confidence of midwives in Ghana to deliver effective bereavement support, and little is known about the personal needs and organisational structures required to enable them to provide such care. This lack of information makes it difficult to determine the specific support midwives themselves need in order to assist bereaved women in recovering physically and emotionally after perinatal loss. This study, therefore, sought to assess the determinants of midwives’ behaviour in providing bereavement support to mothers experiencing perinatal loss in the Upper West Region through the lens of the theory of Planned Behaviours. Guided by the Theory of Planned Behaviour, the study explored midwives’ attitudes, perceived behavioural control, social norms, and behavioural intentions, with the aim of identifying gaps and informing targeted interventions to improve bereavement care practices. By doing so, the study hopes to contribute to better outcomes for bereaved mothers and foster a more compassionate maternal healthcare environment in Ghana.

## Methods

### Study area and period

The study was conducted between February and September 2024 in six districts/municipalities of the Upper West Region of Ghana. The Upper West Region comprises eleven administrative districts with an estimated population of about 901,502. The region was selected because it is one of the most deprived areas in the country and has a relatively high prevalence of perinatal losses. The study focused on public healthcare facilities that provide maternity and related services within the selected districts; Wa Municipal, Wa West, Lawra, Sissala East, Nandom, and Nadowli [18]

### Study design and population

A descriptive cross-sectional quantitative design was employed to assess midwives’ behaviours in providing bereavement support to mothers experiencing perinatal loss. The study adhered to the STROBE guidelines and was guided by the Theory of Planned Behaviour [19]. Participants were registered midwives with at least one year of work experience in public health facilities in the Upper West Region of Ghana, specifically those working in labour wards, antenatal units, lying-in wards, and gynaecological units. Excluded were midwives in medical units not directly involved in maternal care within the past year, as well as interns and contract staff.

### Sample size and sampling procedure

The sample size was calculated using Yamane’s (1967) formula at a 5% margin of error, yielding a minimum of 249 midwives from a population of 662**.** Calculating 10% non-response rate = 274 midwives.

A total of 274 midwives who met the eligibility criteria were included in the study. To ensure fair distribution, the sample was apportioned using a simple proportionate sampling approach, such that districts with larger numbers of midwives contributed proportionately more participants. The final achieved sample exceeded this minimum, enhancing the statistical power and representativeness of the findings.

A multistage probability sampling approach was used. In the first stage, all districts in the Upper West Region with public health facilities providing maternity and related services were identified. Six districts met this criterion and were included as sampling clusters. In the second stage, all public hospitals, health centres, and CHPS facilities within these districts that employed at least one registered midwife were included. In the final stage, a complete enumeration of all registered midwives working in these facilities was conducted. Using Yamane’s formula, a sample size of 249 was required, which was adjusted to 274 to compensate for potential non-response. A total of 269 completed questionnaires (98% response) ultimately received.

### Data collection procedure

Data were collected over a four-week period using a structured, self-administered questionnaire developed from validated Theory of Planned Behaviour constructs. The questionnaire was distributed in paper format to eligible midwives at their respective health facilities during working hours. Trained research assistants explained the purpose of the study and obtained written informed consent before administration. Midwives completed the questionnaire in English, with clarifications provided in local languages when necessary. Completed questionnaires were collected immediately after completion to minimize non-response and ensure data quality.

### Study instrument

The questionnaire also included items on socio demographic characteristics (e.g., age, sex, religion, ethnicity, educational level, years of practice, and unit of work) and professional training exposure related to bereavement care. It was followed by other sections which are Attitude, Subjective Norms, Perceived Behavioural Control, Behavioural Intention, and Bereavement Support Behaviour. Each construct was measured with multiple items adapted from previously validated TPB scales and contextualized for perinatal bereavement support among midwives. The questionnaire used a 5-point Likert scale (1 = strongly disagree to 5 = strongly agree) for all TPB constructs. Minimum possible score = 1.00, maximum = 5.00. There were no cut-off points (continuous data); higher scores simply indicated more favourable attitudes, norms, control, intentions, or behaviours.

The TPB constructs were operationalized using items adapted from Ajzen’s (2006) guidelines for constructing TPB questionnaires. The instrument comprised: Attitude (6 items; e.g., “Providing bereavement support is valuable”), Subjective Norms (4 items), Perceived Behavioural Control (5 items; e.g., “I am confident I can provide effective bereavement support”), Behavioural Intention (3 items; e.g., “I intend to provide bereavement support to bereaved mothers”), and Bereavement Support Behaviour (8 items). Items were adapted by replacing generic health behaviour language with bereavement-specific terminology to ensure contextual relevance. Content validity was established through expert review by three maternal health specialists prior to pilot testing.

Prior to the main study, the instrument was pretested with 20 midwives working outside the study facilities to ensure clarity, cultural appropriateness, and internal consistency. Minor revisions were made based on feedback. The questionnaire was adapted from validated Theory of Planned Behaviour (TPB) scales, contextualized for perinatal bereavement. Content validity was ensured through expert review and pretesting among 20 midwives outside the study sites. Internal consistency was assessed using Cronbach’s alpha after piloting, which ranged from 0.58–0.91.

### Data processing and analysis

Data were entered into Microsoft Excel, checked for entry errors, and exported into IBM SPSS Statistics version 27.0 for analysis. There were no missing values. Descriptive statistics, including frequencies, percentages, means, and standard deviations, were used to summarize participants’ sociodemographic and professional characteristics. For the Theory of Planned Behaviour (TPB) constructs (attitude, subjective norms, perceived behavioral control, behavioral intention, and bereavement support behaviour), item responses were summed and averaged to generate composite scores. Internal consistency of each construct was evaluated using Cronbach’s alpha.

Spearman’s rank correlation was used to examine bivariate associations among the TPB constructs. To test the hypothesized relationships, a path analysis was conducted using AMOS (version 23), with the composite construct scores entered into the model. Model fit was evaluated using multiple indices, including χ²/df, Goodness-of-Fit Index (GFI), Root Mean Square Residual (RMR), Normed Fit Index (NFI), Comparative Fit Index (CFI), Incremental Fit Index (IFI), and Root Mean Square Error of Approximation (RMSEA). The statistical significance of direct and indirect effects was evaluated using bootstrapped confidence intervals (95%). A p-value of <0.05 was considered statistically significant.

### Ethical consideration

Ethical approval for this study was obtained from the Ghana Health Service Ethics Review Committee (GHS-ERC: 059/11/23). In addition, permission was granted by the Ghana Health Service and the respective district and facility authorities. Participation was voluntary, and written informed consent was obtained from all respondents after they were provided with information on the study’s objectives, procedures, potential risks, and benefits. Confidentiality and anonymity were ensured by assigning unique codes to participants, with no personal identifiers collected. All data were securely stored and accessible only to the research team.

## Results

### Sociodemographic characteristics of participants

A total of 269 midwives participated in the study, representing a response rate of 98%. The mean age was 34 years (SD = 5.2, range = 23–55). Participants were predominantly female (97%) and Christian (70.3%). More than half belonged to the Dagaaba ethnic group (52.4%), and the majority held a diploma or post-diploma qualification (70.6%). About two-thirds (68.8%) had ≤ 5 years of work experience (Table 1). More than half (56.9%) reported receiving bereavement support training during their nursing/midwifery education, while about one-third (34.6%) had received in-service training. A large proportion (74.7%) had encountered a woman experiencing perinatal loss in practice. Participants were drawn from various units, with the largest proportions working in antenatal care (40.9%) and labour wards (34.6%) (Table 2).

**Table 1:** Sociodemographic Characteristics of Participants.

Variable	Freq(n)	Percent (%)
**Mean Age ± SD**	34 ± 5.2	
**Age Group (years)**		
20–29	48	17.8
30–39	180	66.9
40–49	37	13.8
50–59	4	1.5
**Sex**		
Female	261	97.0
Male	8	3.0
**Religious Affiliation**		
African Tradition	1	0.4
Christianity	189	70.3
Islam	79	29.4
**Ethnic Group**		
Akan	19	7.1
Dagao	141	52.4
Dagomba	7	2.6
Gonja	13	4.8
Sissala	28	10.4
Waali	51	19.0
Other	10	3.7
**Midwifery Education Level**		
Certificate	14	5.2
Diploma/Post-basic diploma	190	70.6
First Degree	65	24.2
**Years of Experience (Median, IQR)**	4.9 (2–7) —	
**Experience Group**		
≤ 5 years	185	68.8
> 5 years	84	31.2

Standard deviation = SD. Interquartile range =IQR, N= 269.

**Table 2:** Professional Experience and Training Characteristics of Participants (N = 269).

Current Unit/Department	Freq. (n)	Percent (%)
Antenatal Care Unit	110	40.9
Gynaecological Unit	11	4.1
Labour	93	34.6
Lying-In	30	11.2
Neonatal Unit	3	1.1
Postnatal Care Unit	15	5.6
Other	7	2.6
**Encountered Perinatal Loss in Practice**		
Yes	201	74.7
No	68	25.3
**Received In-School Training on Bereavement Support**		
Yes	153	56.9
No	116	43.1
**Received Post-School Training on Bereavement Support**		
Yes	93	34.6
No	176	65.4

### Descriptive statistics of key constructs

Midwives reported generally favourable bereavement support behaviours (Table 3). The highest-rated behaviours were demonstrating empathy and compassion (M = 3.47, SD = 0.86) and providing support for women experiencing perinatal loss (M = 3.41, SD = 0.76). Respecting cultural and religious sensitivities was also rated highly (M = 3.41, SD = 0.82). In contrast, coordinating with social workers, therapists, and counsellors (M = 2.75, SD = 1.22) and providing consistent bereavement support (M = 2.84, SD = 1.13) were less frequently endorsed.

**Table 3:** Midwives’ Behaviour Towards Providing Support to Perinatal Loss Women.

	Min	Max	Mean	Std Dev.
I provide support for women dealing with perinatal loss	1.00	4.00	3.41	0.76
I consistently offer guidance to women dealing with perinatal loss.	0.00	4.00	3.07	0.96
I demonstrate empathy and compassion when interacting with women experiencing perinatal loss.	0.00	4.00	3.47	0.86
I collaborate effectively with other health professionals to provide comprehensive support to women during perinatal loss.	0.00	4.00	3.17	0.93
I provide support based on specific needs of women who experience perinatal loss.	0.00	4.00	3.14	0.96
I accept feedback from women, families and colleagues to improve my support for women experiencing perinatal loss.	0.00	4.00	3.24	0.95
I coordinate with social workers, therapist and counsellors to enhance the support to women experiencing perinatal loss.	0.00	4.00	2.75	1.22
I respect the cultural and religious sensitivities of women whiles providing during perinatal loss.	1.00	4.00	3.41	0.82
I provide consistent and reliable bereavement support to women during perinatal loss	0.00	4.00	2.84	1.13
I maintain a positive and supportive attitude with women in challenging situations during perinatal bereavement support.	0.00	4.00	3.20	1.01

Descriptive statistics and internal consistency of the Theory of Planned Behaviour (TPB) constructs are shown in Table 4. Midwives reported generally favourable attitudes toward providing bereavement support (M = 3.26, SD = 0.41, α = .80). Subjective Norms (M = 3.07, SD = 0.59, α = .91) and Perceived Behavioural Control (M = 3.02, SD = 0.57, α = .87) demonstrated strong internal consistency. Behavioural Intention had a mean of 3.14 (SD = 0.56) but lower reliability (α = .58). Self-reported Bereavement Support Behaviour was relatively high (M = 3.29, SD = 0.49, α = .88). All constructs showed negative skewness, indicating clustering of responses toward higher scores.

**Table 4:** Descriptive Statistics and Internal Consistency of Key TPB Constructs (N = 269).

Variable	Items	A	Mean ± SD	Median (IQR)	Skewness ± SE	Kurtosis ± SE	Min–Max
**Attitude**	10	.80	3.26 ± 0.41	3.25 (3.00–3.58)	–0.52 ± 0.15	0.37 ± 0.30	1.92–4.00
**Subjective Norms**	10	.91	3.07 ± 0.59	3.10 (2.80–3.40)	–1.05 ± 0.15	2.32 ± 0.30	1.00–4.00
**Perceived Behavioural Control**	10	.87	3.02 ± 0.57	3.00 (2.60–3.40)	–0.31 ± 0.15	–0.05 ± 0.30	1.00–4.00
**Behavioural Intention**	3	.58	3.14 ± 0.56	3.00 (3.00–3.67)	–0.48 ± 0.15	0.11 ± 0.30	1.33–4.00
**Behaviour**	10	.88	3.29 ± 0.49	3.20 (3.00–3.70)	–0.56 ± 0.15	0.45 ± 0.30	1.40–4.00

### Correlation analysis

Spearman’s rank correlations (Table 5) indicated significant positive associations among all TPB constructs. Attitude, Subjective Norms, and Perceived Behavioural Control were each significantly correlated with Behavioural Intention and Behaviour. The strongest association was observed between Behavioural Intention and Behaviour (ρ = .75, p < .001), suggesting intention was closely aligned with self-reported practice.

**Table 5:** Spearman’s Rank Correlations Among Key Study Variables (N = 269).

Variable	1	2	3	4	5
**1. Attitude**	**—**				
**2. Subjective Norms**	.52**	**—**			
**3. Perceived Behavioural Control**	.46**	.56**	**—**		
**4. Behavioural Intention**	.40**	.44**	.54**	**—**	
**5. Behaviour**	.49**	.34**	.36**	.75**	**—**

Values are Spearman’s rho correlation coefficients. ** Correlation is significant at the 0.01 level (2-tailed).

### Path analysis

**Fig 1:**
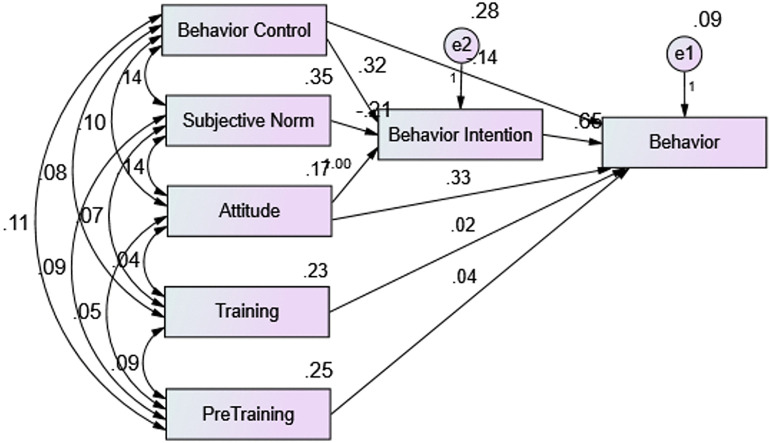
Path diagram showing standardized regression weights for predictors of bereavement support behaviour.

A TPB-based PATH analysis was estimated to examine predictors of bereavement support behaviour (Table 6). The model demonstrated acceptable fit on some indices (GFI = .93; RMR = .05), with moderate incremental fit (NFI = .87; CFI = .87; IFI = .88). However, the χ²/df ratio (21.33, p < .001) and RMSEA (.28, 90% CI:.23–.33) indicated poor overall fit. Standardized regression weights (Table 5) revealed that Attitude (β = 1.00, p < .001), Perceived Behavioural Control (β = .32, p < .001), and Subjective Norms (β = –.21, p < .001) significantly predicted Behavioural Intention, which in turn strongly predicted Behaviour (β = .65, p < .001). Attitude (β = .33, p < .001) and Perceived Behavioural Control (β = –.14, p < .001) also had direct effects on Behaviour. In contrast, pre-bereavement (β = .04, p = .35) and post-bereavement training (β = .02, p = .67) did not significantly predict Behaviour. The Fig 1 illustrates the paths and standardized regression weights between the constructs.

**Table 6:** Standardized Regression Weights from the Path Analysis of Predictors of Bereavement Support Behaviour among Midwives.

Path	Estimate (β)	SE	CR	p
Behavioural Intention ← Perceived Behavioural Control	.32	.06	5.14	< .001
Behavioural Intention ← Attitude	1.00	–	–	–
Behavioural Intention ← Subjective Norms	–.21	.06	–3.53	< .001
Behaviour ← Behavioural Intention	.65	.03	19.05	< .001
Behaviour ← Perceived Behavioural Control	–.14	.04	–3.70	< .001
Behaviour ← Attitude	.33	.06	5.86	< .001
Behaviour ← Pre-Bereavement Training	.04	.04	0.94	.35
Behaviour ← Post-Bereavement Training	.02	.04	0.43	.67

## Discussion

To improve clarity and alignment with the Theory of Planned Behaviour, the discussion has been reorganised according to the five core TPB constructs: (1) attitude, (2) subjective norms, (3) perceived behavioural control, (4) behavioural intention, and (5) actual behaviour

### Attitude

The study demonstrated that midwives’ attitudes were the strongest predictor of behavioural intention and actual bereavement support behaviour. This aligns with previous TPB-based research indicating that personal beliefs about the value and importance of a behaviour are central to shaping professional actions in healthcare settings [19]. In emotionally demanding domains such as bereavement care, positive attitudes particularly beliefs that providing support is meaningful, compassionate, and professionally necessary appear essential for motivating midwives. Evidence from nursing and midwifery literature similarly highlights that professionals’ emotional competence, empathy, and intrinsic motivation influence the quality of bereavement care provided [5,6]. The strong attitudinal effect observed here reinforces the need to cultivate positive professional values through reflective practice, mentorship, and emotionally grounded training approaches.

### Subjective norms

Subjective norms had a statistically significant but comparatively weaker negative effect on behavioural intention (β = –.21). Although significant, this effect was modest next to the much larger contributions of attitude (β = 1.00) and perceived behavioural control (β = .32). This clarifies the earlier description of subjective norms as having a “weaker influence.” The negative coefficient suggests that, in this context, social pressure may actually discourage bereavement support. Midwives may perceive limited endorsement from colleagues, supervisors, or the broader institutional culture phenomena previously reported in Ghana and other low-resource settings [20,21]. Such environments can foster discomfort or avoidance in engaging bereaved families, even when individual attitudes are positive.

The weaker normative influence likely reflects broader sociocultural and organizational constraints in the Ghanaian setting, where bereavement support is often not explicitly expected, supported, or institutionally prioritized. Addressing this requires leadership commitment, role modelling, and systemwide reinforcement to embed bereavement care as a routine and valued aspect of midwifery practice.

### Perceived Behavioural Control (PBC)

Perceived behavioural control showed a dual influence, with a positive effect on behavioural intention (β = .32) and a small negative direct effect on behaviour (β = −.14). This pattern strong prediction of intention but weaker and sometimes opposite prediction of behaviour is consistent with classic and recent tests of the Theory of Planned Behaviour, which find PBC frequently predicts intention but often explains less variance in actual behaviour or shows differing direct/indirect effects [22,23]. On one hand, greater perceived control enhances confidence and motivation, thereby increasing intention to provide bereavement support. On the other hand, the negative direct path suggests that even when midwives feel capable external constraints such as lack of time, heavy workload, or absent institutional protocols may impede their ability to translate intentions into practice [24].

This mismatch between perceived capability and actual behavioural opportunity can generate frustration, weakening the translation of intention into action. Similar findings from bereavement care studies suggest that self-efficacy alone is insufficient unless organizational systems actively support emotionally demanding tasks [25,26]. This underscores the need for institutional reforms that include resource provision, workload adjustments, supportive supervision, and clear bereavement care guidelines. Concretely, our findings indicate that interventions should not target only individual-level confidence (e.g., training) but also system-level change: protected time, bereavement protocols, staffing ratios, and routine debriefing/supervision to reduce the gap between intention and observable bereavement-support behaviours [27].

### Behavioural intention

Intention emerged as a strong predictor of behaviour, consistent with the TPB assumption that intention is the most proximal determinant of action. This finding aligns with meta-analytic evidence showing that intention typically explains substantial variance in behaviour, though rarely enough for full behavioural prediction [28]. However, the strength of intention alone was insufficient to overcome normative and structural barriers. Although many midwives expressed willingness to provide bereavement support, the absence of supportive social norms and system-level enablers reflects what TPB literature calls the “intention-behaviour gap,” particularly pronounced for complex or emotionally demanding tasks. Studies show that even strong intentions fail when the behaviour requires emotional labour, time, or institutional backing [29].

Consistent with this, research on perinatal bereavement care reports that willingness alone does not translate into action without team norms, supervision, and organizational prioritization [30]. This supports the interpretation that behavioural performance in bereavement support is contingent not only on intention but on the presence of shared norms, workload flexibility, and institutional policies that legitimize and support such care.

### Behaviour

Actual bereavement support behaviours were influenced predominantly by attitudes, perceived control, and intention. Training both pre-service and in-service did not significantly predict behaviour, a finding that aligns with broader evidence showing that conventional didactic or short-term training alone often fails to produce sustained changes in clinical practice. For example, a scoping review of continuing professional development (CPD) for health professionals reported that despite knowledge gains, “simply delivering CPD activities … does not lead to a change in practice.” [31] Similarly, a systematic review of training interventions aimed at behaviour change found that while training often improved delivery quality in the short to medium term, the effect on long-term behaviour change was modest (small standardized mean difference), and improvements tended to fade over time when training lacked practical or reinforcement components [32].

The results suggest that enhancing actual practice requires more than “one-off” training: a combination of experiential learning (e.g., role-play, supervised practice), ongoing supportive work environments, clear guidelines, and institutional mechanisms that embed bereavement support into routine maternity care are likely necessary. Indeed, systematic reviews in areas such as palliative care education highlight that many interventions (often including didactic sessions, role-play and coaching) improved healthcare workers’ self-reported confidence, but objective or long-term measures of behavioural change were inconsistent or lacking [33].

In settings where training is not reinforced with structural support – such as time allocation, institutional protocols, supervisory feedback and opportunities for practice – health workers may revert to prior routines. This is particularly problematic for emotionally demanding or low-priority tasks (like bereavement support) that compete with busy workloads. Thus, to translate positive attitudes, intention and perceived control into actual bereavement care behaviour, interventions should target both the individual (skills + self-efficacy) and the system (workload, supervision, institutionalization of bereavement support). This dual approach aligns with recommendations from implementation science and behaviour-change frameworks in healthcare [34].

### Implications for practice and policy

These findings highlight several implications for improving bereavement care in Ghana’s maternal health services:

1. Strengthening Attitudes and Confidence: Educational interventions should prioritize shaping positive attitudes and building self-efficacy in bereavement care by implementing simulation workshops and mentorship programs for building midwives’ confidence in bereavement care.
2. Addressing Normative Pressures: Efforts are needed to reshape workplace cultures so that bereavement support becomes a shared expectation among midwives, supervisors, and health institutions. Health institutions should train supportive leadership and peer networks to help transform norms from potential barriers to facilitators.
3. Revisiting Training Approaches: The lack of significant training effects calls for revising of the midwifery curriculum to include practical workshops on grief, cultural competence in bereavement care, and emotional support strategies. Integrating culturally appropriate communication skills and psychosocial care into both pre-service and in-service programs may improve outcomes. For example, case studies and role-playing exercises.
4. Policy and Systems Support: Develop and implement clear, evidence-based bereavement care protocols. Allocate time in the work schedule for midwives to engage in bereavement care activities, such as providing emotional support and conducting follow-up visits. Ensure that midwives have access to the necessary resources, such as counselling services, to offer comprehensive support.

### Strengths and limitations

A major strength of this study is its use of a theory-driven framework (TPB), which provides a robust basis for examining psychosocial predictors of midwives’ behaviour. The relatively large sample size and high response rate also enhance the reliability of findings.

However, limitations must be noted. The cross-sectional design precludes causal inferences, and the reliance on self-reported behaviour introduces the possibility of social desirability bias. Additionally, the study was conducted in a single region of Ghana, which may limit generalizability to other settings with different cultural or institutional contexts. The low internal consistency of the behavioural intention scale (α = .58) also warrants caution in interpreting that construct. Construct validity was not assessed through factor analysis, limiting our ability to confirm whether adapted items loaded appropriately on their intended constructs. Second, the Behavioural Intention scale demonstrated lower internal consistency (α = .58) compared to the conventional.70 threshold, which may have affected the precision of path estimates involving this construct and warrants cautious interpretation.

## Conclusion

The findings demonstrate that attitudes and perceived behavioural control are central predictors of both intention and behaviour, while subjective norms may function as barriers in this context. The findings emphasize the need for improvements in bereavement care by focusing on enhancing midwives’ confidence, fostering supportive institutional norms, and revising training curricula to include culturally sensitive, practical skills by policymakers, researchers and educators. By addressing these gaps, maternal health services in Ghana can improve the quality of support provided to women and families experiencing perinatal loss, ultimately contributing to more compassionate and holistic maternity care.
